# A Simple low-cost device enables four epi-illumination techniques on standard light microscopes

**DOI:** 10.1038/srep20729

**Published:** 2016-02-08

**Authors:** Robert R. Ishmukhametov, Aidan N. Russell, Richard J. Wheeler, Ashley L. Nord, Richard M. Berry

**Affiliations:** 1 Department of Physics, Clarendon Laboratory, University of Oxford, Parks Road, Oxford, OX1 3PU, UK; 2 The Sir William Dunn School of Pathology, University of Oxford, South Parks Road, Oxford, OX1 3RE, UK

## Abstract

Back-scattering darkfield (BSDF), epi-fluorescence (EF), interference reflection contrast (IRC), and darkfield surface reflection (DFSR) are advanced but expensive light microscopy techniques with limited availability. Here we show a simple optical design that combines these four techniques in a simple low-cost miniature epi-illuminator, which inserts into the differential interference-contrast (DIC) slider bay of a commercial microscope, without further additions required. We demonstrate with this device: 1) BSDF-based detection of Malarial parasites inside unstained human erythrocytes; 2) EF imaging with and without dichroic components, including detection of DAPI-stained *Leishmania* parasite without using excitation or emission filters; 3) RIC of black lipid membranes and other thin films, and 4) DFSR of patterned opaque and transparent surfaces. We believe that our design can expand the functionality of commercial bright field microscopes, provide easy field detection of parasites and be of interest to many users of light microscopy.

Modern light microscopy offers a variety of sophisticated techniques which require either expensive and bulky additions to commercial microscopes or development of custom-built microscopes that require expertise in optics and opto-mechanics. Back-scattering Dark Field (BSDF), epi-fluorescence (EF), interference reflection contrast (IRC) and surface reflection (SR) fall into this category. BSDF is not yet commercially available, and offers greatly enhanced contrast for small objects relative to larger scatterers, compared to commercially available forward-scatter darkfield^1^. BSDF also allows free access to the sample by removing the need for a conventional darkfield condenser. Applications include observation of metallic nanoparticles against the background of bacterial or animal cells^1^,^2^,^3^ and detection of nano-crystals of the pigment hemozoin in human erythrocytes infected by malarial parasites^4^. EF is the basis of numerous biomedical and public health applications, including field detection of many epidemiologically relevant infections^5^,^6^. RIC is used in the study of thin films, for example in the biological sciences to study cell-surface interactions and dynamics of lipid bilayers^7^. SR, which is indispensable in materials science and numerous industrial applications, including its dark field version (DFSR), traditionally needs special objectives to study opaque materials^8^.

Simplification, miniaturization and cost reduction are of interest to expand the availability of these techniques to non-specialist users, including those outside the research community. To address these challenges we have developed a prototype device which combines these four techniques in a simple, small, low-cost attachment. The device inserts into the differential interference-contrast (DIC) slider bay of a commercial microscope to deliver extra light-paths for the four techniques, with no further additions to the microscope. Illumination is delivered through an optical fibre and a small mirror mounted in the device, just below the microscope objective. The device can be removed to enable the commercial imaging mode of the microscope and quickly inserted to enable imaging of the same sample in one of the above modes, for example epi-fluorescence imaging - in certain cases without the need for any dichroic filters (saving significant costs). Commercial fluorescence microscopes can be enhanced using our device as a secondary illumination path, for example by combining darkfield with fluorescence in the same image.

## Results

### Design of the device

**Figure 1:**
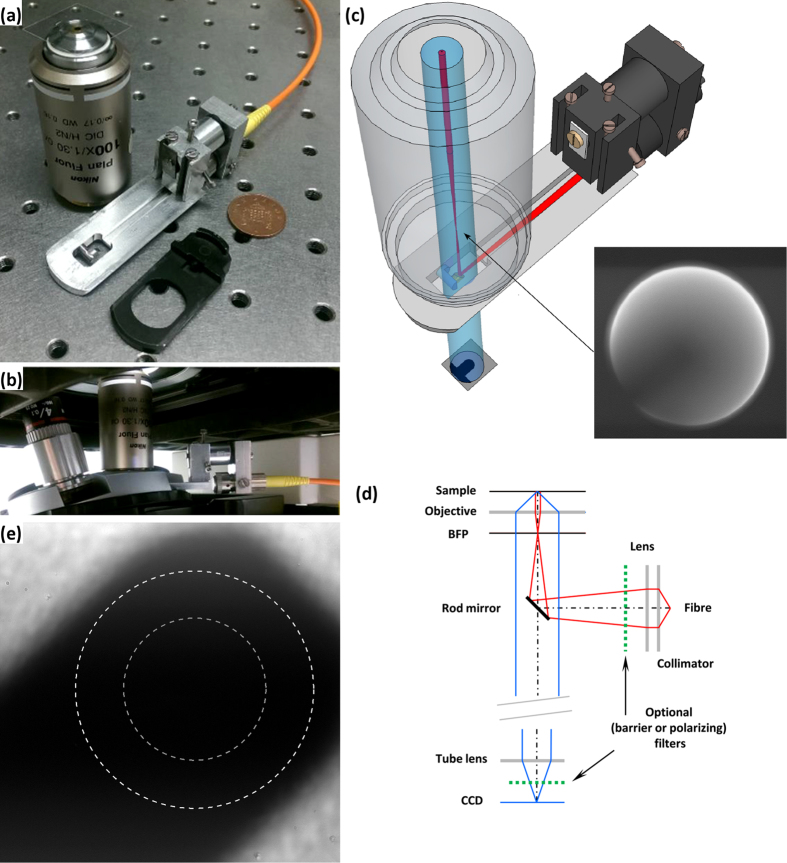
Prototype epi-illuminator device. (**a**) The device (**middle**) is compared for size to a UK 1 pence coin (**right**) and a Nikon 100 × oil immersion objective (**left**). Light is coupled into the objective by a small mirror mounted on the “insert” part of the device which matches the shape of a Nikon “DIC slider” (**bottom**). The remainder of the device consists of a mount for an optical fibre and lens, which focuses illumination light onto the mirror. (**b**) The device mounted on an inverted Nikon research microscope. (**c**) A schematic of the device. The inset shows an image of the 100 × NA 1.45 objective’s back focal plane with the out-of-focus shadow of the 2 mm rod mirror. This comes from 200 nm fluorescent beads stuck on the coverslip surface, in water, objective is epi-illuminated. (**d**) Ray diagram of the device in back scattering dark field and epi-fluorescent modes. Incident rays (red) reflected by the rod mirror into the objective and reflected back by the planar surfaces (the coverslip and the slide) are blocked by the mirror. Light scattered or emitted by the specimen (blue rays) passes the mirror and forms the image. Optional filters (green) may be used to enhance the signal and remove the background. (**e**) 100 × objective field of view showing the shadow formed by the 2 mm rod mirror with bright field illumination of NA = 0.52. White and grey dotted circles, 50 and 30 μm in diameter, indicate the size of the illumination spot formed by LEDs and laser, coupled through multimode and single-mode fibres respectively in our prototype.

The key to epi-illumination techniques including those achieved by our device is to separate the signal (fluorescence, scattered, phase- or polarization-shifted light coming back through the objective toward the detector) from the background (scattered light in the case of fluorescence and in all cases scattering or reflection from interfaces external to the sample, for example between the coverslip and sample volume). Our device (patent pending) performs this separation spatially in a plane near to the back focal plane of the objective, accessed by matching the shape of the device to commercial Wollaston Prism mounts (“DIC sliders”) which fit the objective prism slot (often called DIC objective slot) of a research microscope. Our prototype device consists of two parts, “insert” and “mount” (Fig. 1A,B). The insert holds the key component of the device: a small horizontal 45° angled rod mirror positioned with its centre on the optical axis of the objective. The mount in our prototype was designed to provide lateral translation, focus and tilt of the illumination beam.

The mirror plays a double role. First, it provides epi-illumination of a specimen by reflecting a narrow beam of light, emerging from a lens and optical fibre on the mount, along the optical axis. The illumination beam is focused to cast an image of the fibre output face onto the back focal plane (BFP) of the objective, giving Köhler illumination of the specimen (i.e. light from each point of the fibre is collimated at the specimen) (Fig. 1C,D). In general, any combination of fibre and illumination source could be used. Second, the mirror blocks illumination reflected directly by planar surfaces, particularly those of any coverslip or slide used to mount the sample. If the mirror were placed in the back focal plane of the objective, it would be possible to block all such reflected light. However the DIC slot is typically a few cm from the objective back focal plane (~50 mm in our setup), in which case reflected light is blocked only within a shadow cast by the mirror (Fig. 1C,E). The size of the shadow depends on the size of the mirror and the size and collimation of the illuminating beam. In our prototype the mirror is 4 mm long by 2 mm in diameter or 2 mm long by 1 mm in diameter (mirrors of such sizes are the smallest rod mirrors available commercially), and the shadow covers a region 160 × 60 or 80 × 30 μm respectively within the 220 μm field of view of a 100 × objective under Köhler bright field illumination.

In this study we used a commercial inverted Nikon microscope equipped with 100 × Nikon Plan Fluor objectives with adjustable numerical aperture (NA 0.5–1.3), and a TIRF objective (NA 1.45); all images presented here were collected with these objectives unless otherwise specified.

We used a range of high-power light emitting diodes (LEDs, 300–600 mW) coupled into a 50 μm-core multimode step index fibre, or a 632 nm laser (10 mW) coupled with a single mode fibre. Illumination intensity measured at the sample was 15.3 mW/mm^2^ and 960 mW/mm^2^ for LED and laser respectively. We used aspheric lenses of focal length 11 mm and 4.6 mm to collimate the laser and LED illumination respectively, and a lens of focal length 100 mm to focus the illumination onto the objective BFP. These components provided illumination with NA=0.28 and a circular field 50 μm in diameter for LEDs, and NA=~0.015, 30 μm field diameter for the laser (dashed circles, Fig. 1E).

For quantitative characterization of the performance of the microscope with our device we used an advanced digital camera (Andor iXon Ultra 888, list price ~£25,000), and to demonstrate the low-cost potential of our device we used much cheaper color video (Watec 0.3 mega pixel analogue model WAT221S, ~£200) and digital (Aptina 5 mega pixel digital model DK5.1P, ~£1300) cameras.

The mechanical stability of the illuminating beam was limited by the fit of the insert in the DIC slot, which we did not attempt to quantify. We imaged in wide-field mode only, and never detected any movement or fluctuation of the illumination field under normal viewing conditions, indicating that the mechanical stability was adequate. The insert was made of aluminium, chosen for ease of machining and low thermal expansion for a given quantity of heat absorbed. We did not detect any effects of thermal expansion in our experiments, all of which were carried out at room temperature.

### Optical Resolution

**Figure 2:**
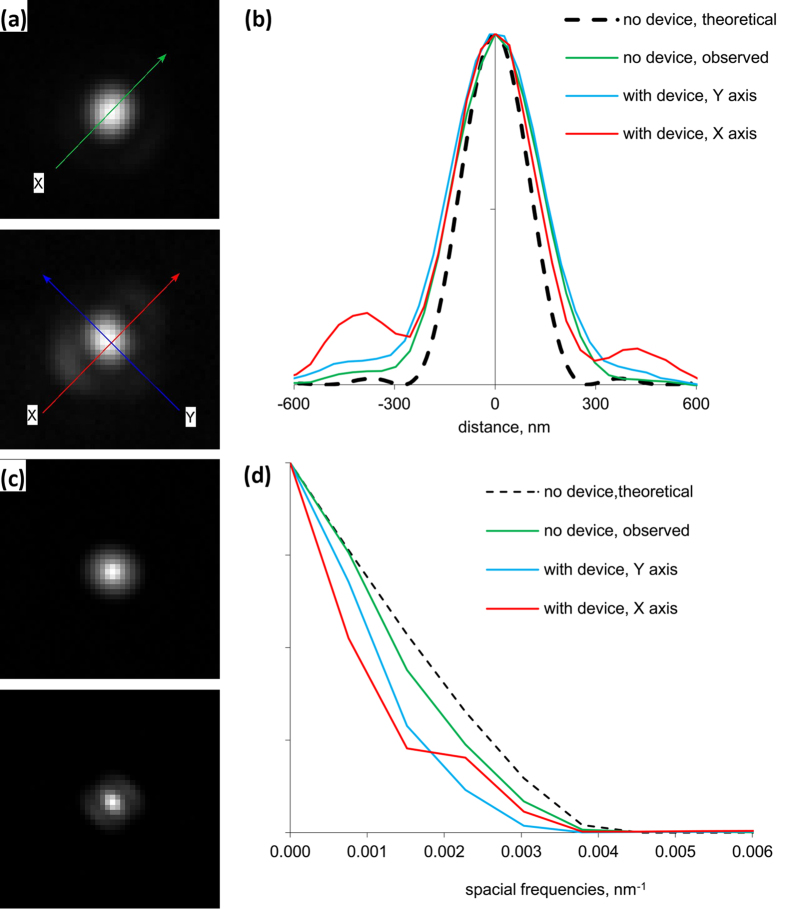
Point spread function and modulus transfer function of the microscope with our device inserted into the DIC slot. (**a**) Point-spread functions (PSF), approximated by fluorescence images (40 nm/pixel) of a 20 nm quantum dot, without (**top**) and with (**bottom**) the device in the DIC slot. We used a custom-built white light TIRF illumination system, a commercial fluorescence filter cube and 1.45NA 100 × objective. (**b**) Sections of the PSFs along the axes shown in A, without the device (green), with the device, along the long (y, blue) and short (x, red) axes of the rod mirror mount, and for comparison the section of the theoretical PSF with no device (dashed black). (**c**) Modulus transfer functions (MTFs) of the images shown in A. (**d**) Sections of the MTFs from C along the same axes as in B, and of the theoretical MTF. The device lowers resolution slightly, and asymmetrically, particularly at intermediate spatial frequencies along its short axis.

The out-of-focus shadow of the rod mirror of our device in the TIRF objective back-focal-plane (BFP) is shown in Fig. 1C, inset. To ensure filling of the BFP we imaged a dense layer of 200 nm diameter fluorescent beads stuck to a coverslip surface, in water, with conventional epi-fluorescence. The bright circle in the image delineates NA 1.33, corresponding to light coupled from the beads into the objective via water, while the dimmer surrounding ring is formed by light coupled directly into the coverglass and delineates the full 1.45 NA of the objective. The shadow of the mirror constitutes an optical mask in the BFP, which reduces the resolution and breaks the axial symmetry of the optical system. This is illustrated in Fig. 2, which compares point-spread functions (PSFs) and modulus transfer functions (MTFs) with and without the device inserted to theoretical PSF and MTF. The device expands the PSF slightly, and asymmetrically, in the manner expected: some spatial frequencies are lost in the MTF, but the full-width at half-maximum (FWHM) of the PSF is essentially unchanged. The PSFs in Fig. 2 were approximated by highly magnified (40 nm/image pixel) fluorescence images of 20 nm quantum dots under conventional EPI-fluorescence illumination. LED EPI-fluorescence illumination with our device gave the same results (data not shown), with slightly reduced brightness.

### Back-scattering Dark Field

BSDF was developed recently and is not yet available commercially. Previously described prototype BSDF microscopes require either major modifications of commercial microscopes^2^,^9^ or development of custom-built microscopes^1^. Two approaches to sample illumination have been utilized in BSDF setups: a large 45° mirror with a small central hole through which the beam illuminates a sample^2^, or a small central mirror either deposited on a flat glass surface^2^,^10^ or consisting of a small 45° angled rod mirror^1^. The last of these approaches is utilized in our device (Fig. 1D). Dark field imaging is observed in the shadow of the rod mirror, where light rays reflected from planar surfaces (the coverslip and slide) are blocked by the mirror, while light scattered by the specimen passes the mirror to form the image. Reflected light misses the mirror only if it originates far from the optical axis (in our case this is light emitted from the periphery of the optical fibre), and forms an interference image beyond the mirror’s shadow (IRC technique, discussed below). The further from the optical axis the light is emitted, the more likely it is to miss the mirror after reflection, encroaching further into the shadow region. In our LED-coupled prototypes a region of full shadow corresponding to darkfield imaging was present with optical fibres 50 μm or less in diameter as long as illumination NA at the objective remained less than 0.3 and 0.15 for 2 and 1 mm rod mirror, respectively.

BSDF offers superior contrast^1^ for small objects against a background of larger objects, compared to the well-established and commercially-available forward scattering dark field (FSDF) microscopy. This is because larger objects scatter light mostly in the forward direction, while objects smaller than the wavelength of light, such as gold nanospheres, approximate to Rayleigh scatterers and scatter more evenly in all directions^1^,^11^. BSDF in an inverted microscope leaves the sample accessible from above, while FSDF using a darkfield condenser, especially at high numerical apertures, drastically restricts access to the sample. Also, our BSDF setup does not depend on low NA objectives in contrast to FSDF, allowing more light from the sample to be collected, and higher resolution imaging.

**Figure 3:**
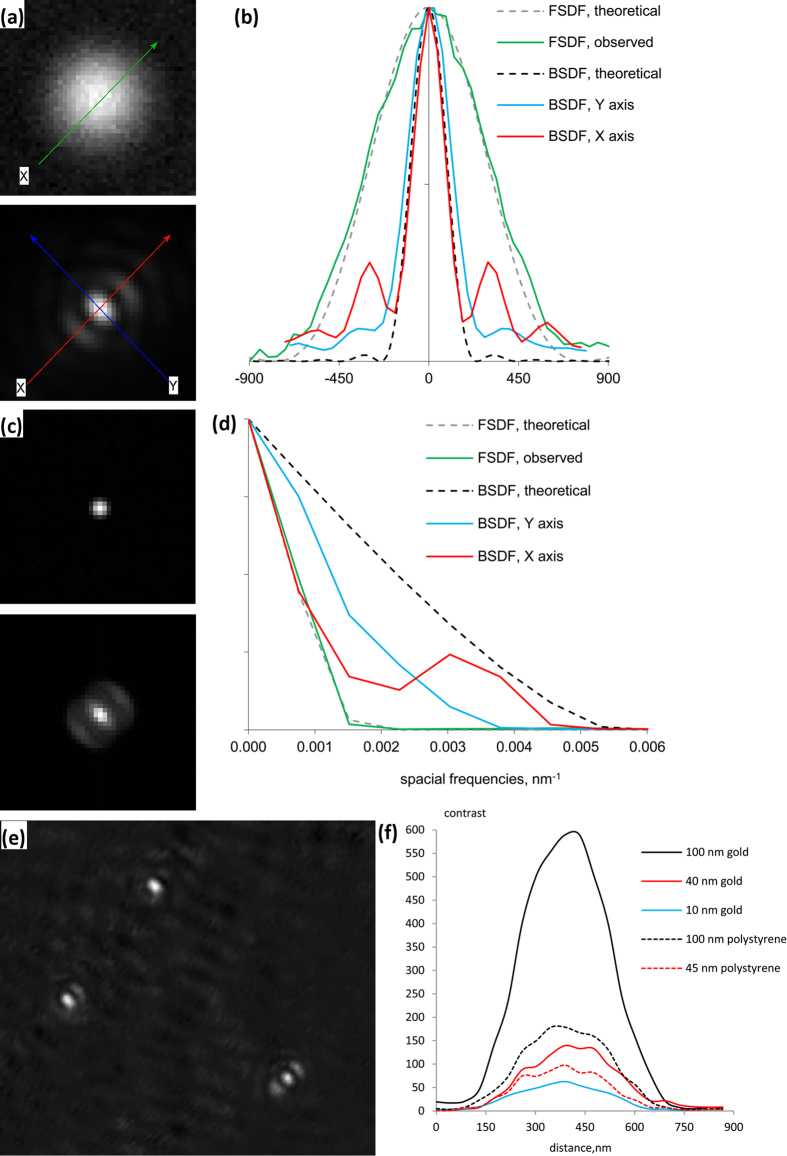
Performance in Back Scattering Dark Field (BSDF) mode compared to commercial Forward Scattering Dark Field (FSDF). (**a**) Dark-field images (40 nm/pixel) of a single 100 nm gold bead using commercial FSDF (top, 100 × objective, NA = 0.5; illumination NA = 0.95−0.75, white light) and BSDF with our device (bottom, a 100 × objective, NA = 1.3; illumination by a 470 nm blue LED via a 50 μm multimode optical fibre). (**b)** Sections of the images in A along the axes shown, and for comparison sections of the theoretical PSFs for unobstructed NA = 0.5 and NA = 1.3 are shown (dashed grey and black). (**c**) Modulus transfer functions (MTFs) of the images shown in A. **(d)** Sections of the MTFs from C along the same axes as in B, and of the theoretical MTFs. The device offers considerably higher resolution than the commercial FSDF system, due to the possibility of observing with high NA. (**e**) BSDF image of 10 nm gold spheres imaged with 632 nm laser via a single mode fibre. (**f**) Contrast (maximal bead pixel value/average background pixel value) for BSDF images of 100 (black), 40 (red) and 10 nm (blue) gold spheres and 100 (dotted black) and 45 nm (dotted red) polystyrene beads.

We compared performance of our device in BSDF mode to a FSDF commercial setup using 100 nm gold nanoparticles. For FSDF we used a commercial dry dark field condenser, (illumination NA 0.95–0.75), 1 mm working distance, and the 100 × variable NA objective set to NA= 0.5. For BSDF illumination we used a 50 μm diameter multi-mode optical fibre coupled to 470 nm LED. Fig. 3A–D shows PSFs and MTFs for the two dark-field modes. The FWHM of the PSF is nearly 3 times less in BSDF than in the commercial setup, illustrating the advantage of the compatibility of BSDF with high NA objectives.

Modern advanced techniques based on back-scattering allow unprecedentedly high contrast imaging of very small structures including 100 nm isolated influenza viruses^11^ (BSDF), 20 nm lipid bilayer domains^12^ and even individual protein molecules^13^ (back scattering combined with interferometry). Our simple device also allows high contrast imaging of very small scatterers. Fig. 3E shows a typical field of view of 10 nm gold spheres imaged with our device coupled to the red laser. Using this configuration of the device we observed 10, 40 and 100 nm gold particles with 1:60, 1:140 and 1:600 contrast (maximal bead pixel value / averaged background pixel value), and 45 and 100 nm polystyrene beads with 1:100 and 1:180 contrast (Fig. 3F). Performance of the commercial FSDF setup was much worse; for example 100 nm gold spheres were seen with 1:6 contrast, 100 times less than BSDF (data not shown), and the weaker scatterers could not be distinguished.

Food vacuoles of *Malaria* parasites living inside host erythrocytes contain crystals of the brown pigment hemozoin^14^, which can reach a few hundred nm in size^15^. The parasite forms hemozoin by depositing free heme, a product of hemoglobin decay which is toxic to the parasite, thus sequestering it from its cells. Due to its birefringence and excellent light scattering hemozoin has been suggested as a promising target for non-invasive fast detection of malaria in unstained blood smears by cross-polarizing (xP) microscopy^16^ and FSDF^17^,^18^. FSDF gives relatively low contrast, with a signal-to-noise ratio (SNR=maximal hemozoin pixel value/maximal empty erythrocyte pixel value) around 1.5–3, which does not allow clear identification of infected cells in crowded blood smears^4^,^19^. xP microscopy is more sensitive than FSDF^20^, though polarizing filters are relatively expensive. An alternative advanced BSDF method was reported to increase SNR up to 50^4^,^19^, which opens up the possibility of automatic detection of the infection. This method combines xP imaging with a sophisticated DFSR system with sample illumination angle varying between 15–45°. Another non-invasive approach includes interferometric tomographic phase microscopy of infected erythrocytes, though this technique is computationally demanding^21^.

**Figure 4:**
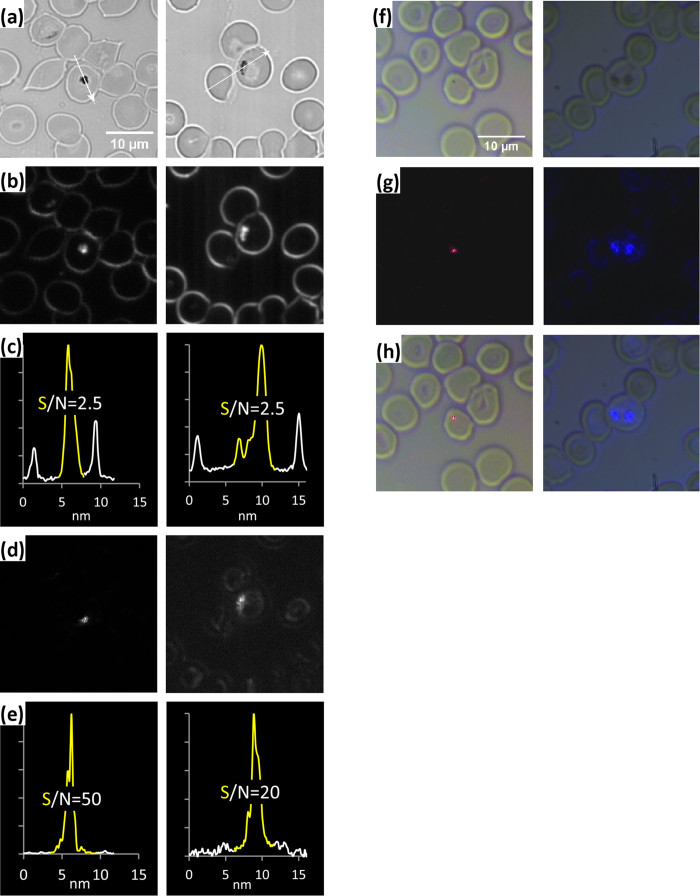
Comparison of our device in BSDF mode with a commercial FSDF setup in detection of *Malaria* parasite inside unstained human erythrocytes. Images were collected with a digital Andor iXon Ultra 888 (**a**–**e**) or analogue color Watec WAT-221S (**f**–**h**) cameras. (**a**) Bright field images of *Malaria falciparum* trophozoite in erythrocytes seen with 1.3 NA 100 × objective. Arrows show the locations where intensity profiles were taken from dark-field images of the same sample fields, shown in B and D. (**b**) FSDF images of the same two fields of view as in A with 0.5 NA 100 × objective and 0.65–0.95 NA illumination. (**C**) Signal-to-noise ratios (SNRs) for FSDF, estimated as the ratio of the peak intensity of the hemozoin crystal (yellow) to that of the erythrocyte membrane (white). (**d**) The same fields of view seen in BSDF with our device coupled to the red laser and equipped with cross-polarizing filters (left), or to the blue LED without polarizing filters (right). (**e**) SNRs calculated as in C for BSDF. (**f**) Bright field image of *Malaria,* (**g**) BSDF image of the same field of view imaged as described for D. (**h**) Combination of BSDF and bright field illumination.

We compared performance of our device in BSDF mode with a commercial FSDF setup in malaria detection by imaging a preparation of unstained human red blood cells, infected with *Malaria falciparum* at trophozoite stage, formaldehyde-fixed, and mounted in isotonic solution. Figure 4A shows two fields of view each containing a single infected cell in bright-field illumination. Figure 4B shows the same fields using commercial FSDF (objective NA = 0.5), with SNR ~ 2.5 (C). For comparison, xP imaging of the same sample gave SNR ~ 10, which was reduced to ~5 when we attempted to combine it with FSDF (data not shown). Figure 4D shows the same fields imaged in BSDF with our device, using laser illumination combined with xP (left, polarizers in front of laser and camera, SNR ~50) or LED illumination at 470 nm without xP (right, SNR ~20). Laser illumination without xP gave SNR ~ 20 (data not shown), similar to LED illumination and previous reports^4^. Figure 4F–H show two infected cells in bright field (F), BSDF as in D (G), and both imaging modes combined (H), using an inexpensive color video camera.

### Epifluorescence

Canonical epi-fluorescent setups include a source of excitation light and a filter cube containing either two filters (a dichroic mirror and an emission filter for narrow-band excitation by lasers or LEDs) or three filters (an additional emission filter for broad-band excitation by mercury or xenon lamps). Our device can be used instead of a filter cube in epi-fluorescent applications when complimented by a narrow-band LED for fluorescence excitation. In case of weak back scattering by the sample our device can be used in BSDF mode for fluorescence without any filters, since reflected excitation light is blocked by the mirror. In case of strong back scattering by the sample, a single emission filter but no dichroic mirror is needed for fluorescence imaging. We tested these EF modes of our device on fluorescent polypropylene beads as well as two microorganisms, a parasite *Leishmania mexicana* and a unicellular green alga.

**Figure 5:**
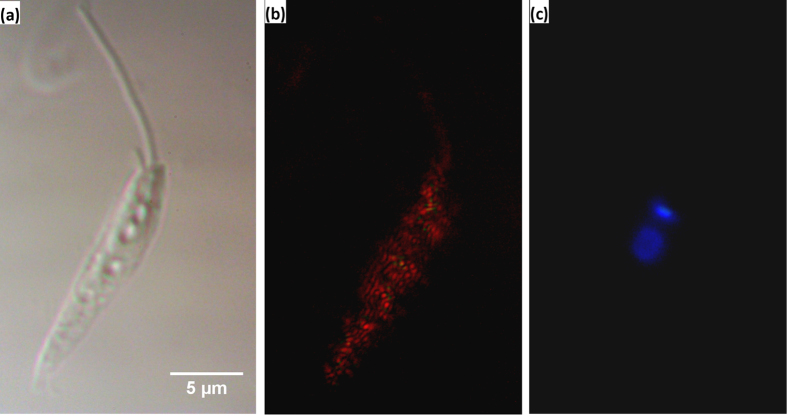
Epi-fluorescent filter-free technique utilized in our device. **(a)** DIC image of *Leishmania mexicana* promastigote seen with 100 × objective. **(b)** BSDF image of the same field of view seen with our device coupled to a warm white light LED via 50 μm multi-mode optical fibre. **(c)** Epi-fluorescent image of the same field of view seen with our device coupled to the 380 nm UV LED, which was used to visualise DAPI - labeled nucleus and kinetoplast of *Leishmania*.

**Figure 6:**
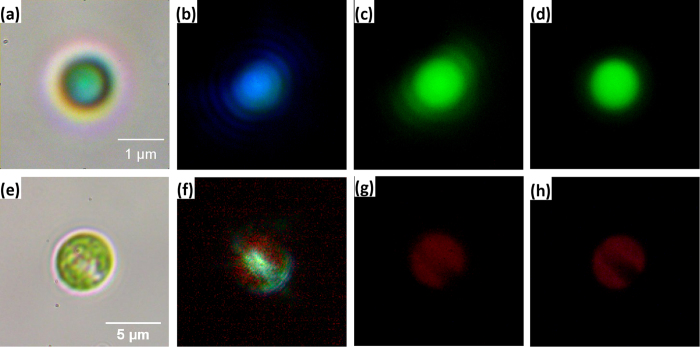
Epi-fluorescence imaging of strongly back-scattering objects by our device complemented by an emission filter. 1 μm green fluorescent polypropylene bead (**a**–**d**) and green alga (**e**–**h**). (**a**,**e**) Bright field image of the samples. (**b**,**f**) Back scattering by the samples illuminated by blue excitation light of the 470 nm LED coupled to our device via 50 μm multi-mode optical fibre and imaged by our device without an emission filter. (**c**,**g**) Same field of view imaged with the emission filter. (**d**,**h**) Same field of view imaged with a commercial filter cube set and a mercury arc lamp.

Promastigotes of *Leishmania* (Fig. 5) have very weak back scattering when illuminated with a broad-band “warm white” light LED (Fig. 5B), and weaker still with a narrow-band 365 nm UV-LED. Figure 5C shows a fluorescence image of the same cell as Fig. 5A,B, using the UV-LED and no filters. The blue spots are the brighter kinetoplast (*Leishmania* mitochondrion) and the dimmer nucleus, labeled with DNA-intercalating blue fluorophore DAPI. As previously shown^22^, DAPI binds the A-T rich kinetoplast DNA better than G-C rich nuclear DNA. Figure 6 illustrates fluorescence imaging using our device with and without filters. With no filters the fluorescence emission from strongly scattering 1 micron green fluorescent beads (Fig. 6B) and a moderately scattering green alga (Fig. 6F) is either overwhelmed or matched, respectively, by the backscatter component. Placement of a suitable emission filter in front of the camera removes backscatter and gives a clear green image of the fluorescent bead (Fig. 6C), or a red fluorescence image of the alga due to its chlorophyll (Fig. 6G). A commercial epi-fluorescence setup (3-filter fluorescent cube and a mercury arc lamp) gives similar images with slightly improved resolution (D, H), after matching the arc lamp illumination intensity (which can be up to 40 times brighter) with the intensity of the LEDs.

### Interference Reflection Contrast (IRC)

**Figure 7:**
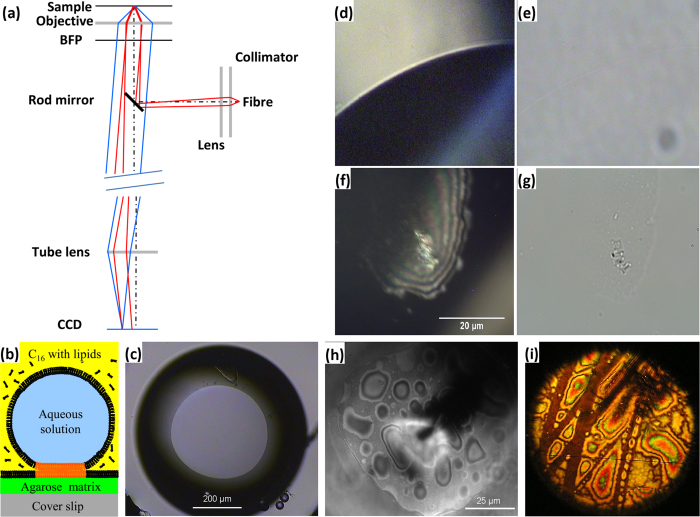
Interference reflection contrast technique (IRC) utilized in our device. **(a)** Ray diagram of IRC. Peripheral rays (red) from near the edge of the fibre reflected by the rod mirror into the objective under larger angle are reflected back by planar interfaces and pass the mirror, to interfere at the camera with light reflected from other surfaces in the sample (blue) thus forming an interference image in a zone surrounding the shadow of the mirror. **(b)** Schematic of the Droplet on Hydrogel Bilayer (DHB) technique. DHBs are formed by contacting two lipid monolayers self-assembled in a hexadecane/lipid mixture at the interface between an aqueous droplet and a thin agarose matrix. **(c)** Bright field image of the bilayer imaged with 5x objective (bottom view). **(d,f)** The edge of a well-formed bilayer **(d)** and a thicker defect **(f)**, imaged with a warm white light LED coupled to the 2 mm rod mirror prototype of our device via 50 μm multi-mode fibre. **(e,g)** the same regions of the bilayer seen in bright field are of much lower contrast;. **(h,i)** Interference zone is much larger for 1 mm rod mirror and 50 μm fibre**. (h)** Transient thicker patches in the lipid bilayer during formation of DHB. **(i)** Uneven deposition of ink material in a marker stripe on a cover slip surface.

This technique is used to study close inter-surface interactions like cell adhesion or lipid bilayer dynamics. Modern setups require a special objective with a built-in quarter-wave plate and a filter cube with two cross-oriented polarizers and a semi-reflecting mirror^7^,^23^. The image is formed by interfering light paths which have been reflected by two surfaces separated by less than the coherence length of the illuminating light, typically of order 1 micron or less; such as glass/medium, medium/membrane, membrane/cytoplasm etc. This technique is quantitative for monochromatic illumination, while usage of wide spectrum illumination without the quarter-wave plate is qualitative and provides an iridescent interference pattern. In our device, unlike BSDF and EF where image formation depends on illumination paths close to the optical axis, image formation in IRC depends on peripheral rays emerging near the edge of the fibre and missing the mirror after reflection from planar surfaces in the sample. These form the interference image outside the shadow cast by the mirror (Fig. 7A).

We tested the performance of our device in IRC on lipid bilayers formed by the Droplet on Hydrogel Bilayer (DHB) method. DHB is a new type of model lipid bilayer^24^ with hugely improved stability compared to previous black-lipid membranes and the additional advantage of allowing observation of the bilayer with high numerical aperture optics^25^. DHBs are formed by contacting lipid monolayers self-assembled in a hexadecane/lipid mixture on the surface of a sub-mm aqueous droplet and a sub-micron agarose layer (Fig. 7B); such bilayers cover few thousand μm^2^ (C). Figure 7D shows the edge of a DHB imaged in IRC with our device with 2 mm mirror and 50 μm-core multimode fibre coupled to the warm white light LED. The lipid bilayer, lower right, has a classic “black lipid membrane” appearance due to destructive interference, in contrast to the area where the bilayer is not formed. Figure 7E shows the same field of view in bright-field, illustrating clearly the superior ability of our device to distinguish the bilayer from non-bilayer area. Figures 7F, G show IRC and bright-field images respectively of a region of the DHB where a thin lens of oil is trapped between the lipid monolayers, constituting a thicker patch between the reflecting interfaces. Again, the enhanced contrast of our device in IRC mode is clear.

The fractions of the field of view represented by the dark field and interference zones depend on the relative sizes of the mirror and the beam. For the larger rod mirror and a small fibre core (2-mm-diameter and 25 μm fibre core) the interference area does not exist because all peripheral rays are blocked by the mirror (data not shown), while for a 1 mm mirror and 50 μm fibre core the interference zone is substantially larger than the dark field area. The latter case is shown in Fig. 7H, I, where in the DHB technique transient thicker patches (H) presumably represent a thickening layer of hexadecane oil trapped between lipid monolayers in the course of the bilayer formation. Similarly, a black ink mark on a glass surface shows pronounced interference due to its uneven thickness (I). The shadow of the mirror is barely visible extending from the top right of these images.

### Dark Field Surface Reflection

This epi-illumination technique is used in industrial applications to visualize relief features of opaque surfaces. Commercial DFSR uses a special reflected-light dark field mirror cube and industrial (metallurgical) objectives^8^,^26^. Such objectives are constructed to accommodate an additional light path inside the objective barrel to form oblique hollow cone illumination of the surface and are designed to be used on dry surfaces, without coverslip or immersion oil. Regular transmitted light objectives used in life sciences research are not suitable for DFSR applications.

**Figure 8:**
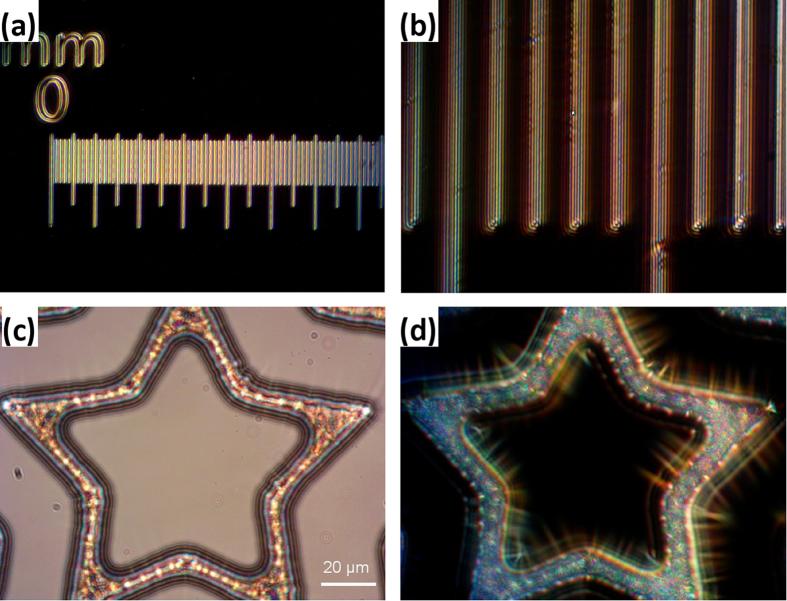
Dark field surface reflection technique (DFSR) by our device and transmission light objectives used without the coverslip. (**a**,**b**) DFSR of the scale of the opaque object micrometer imaged with our device coupled to a warm white light LED via 50 μm multi-mode optical fibre and 5x objective and 40 × objective with additional 1.5x magnification. The distance between the scale bars is 10 μm. (**c**,**d**) Bright field and DFSR images of transparent silica surface etched by photo-lithography as seen with 40 × objective.

Due to its design our device can be used in DFSR applications on regular transmitted light microscopes equipped with regular transmitted light objectives instead of industrial objectives, to test the surface of interest without a coverslip. The configuration of our device for DFSR is essentially the same as for LED-coupled BSDF, the difference being that the sample is a reflecting surface rather than small scattering objects against a transparent background. As in BSDF, rays reflected by large planar surfaces are blocked by the mirror whilst rays scattered by and / or reflected by surface features bypass the mirror and form the image (Fig. 1D). We found that in DFSR mode our device can work with objectives of various magnifications. For example, the 10 μm spaced lines of an opaque object micrometer are clearly seen with all transmitted light objectives (5–40 × magnification) tested (Fig. 8A,B). Transparent surfaces such as photoresist patterns on silica wafers can also be studied with our device (C,D).

## Discussion

This study demonstrates how our novel miniature device can expand the functionality of a conventional bright field microscope by combining four epi-illumination techniques in a simple optical design. Our device can substitute for much more expensive advanced hardware while still providing good performance with minimal loss of resolution. Refinements of the design to minimize masking by the support for the illuminating 45° mirror would further reduce the loss of resolution. The device can expand applicability of these techniques, which may be of special importance for some public health field applications. Cheap, fast and stain-free BSDF-based detection of malaria, which causes a few hundred million clinical cases and kills more than a million people per year around the globe^27^ is an example of such a potentially important application. Similar to a previously published sensitive method^4^, our device provides sensitive detection of malarial parasites with the same signal to noise ratio but without using sophisticated equipment, and even makes it possible to use the device under bright field illumination. Such a high SNR is possible due to low loss of the signal: we collect nearly all back scattered light with a 100 × NA 1.3 immersion objective, and only 5 or 20% of scattered light is blocked by 1 mm or 2 mm mirror, respectively, while in the reported study a lower proportion of scattered light is collected with a lower NA 40 × objective and two polarized filters. To further reduce costs, the BSDF principle of our device could be realized without a research microscope in a simpler device retaining only the essential components: sample holder, objective, mirror, light source and fibre. A similar approach was demonstrated recently with a simple 3D printed iPhone-based xP microscope for malaria detection^28^. Our device may also be of interest for detection of other intracellular pathogens, in particular those with strong back-scattering properties.

Our device also works as a simple filter-free epi-fluorescence system, which also may be valuable for field applications. We tested its applicability on another epidemiologically important parasite – *Leishmania* - which affects more than 10 million people^29^. Fluorescently-labeled cells of this parasite, having a low level of back scattering, were visualized with a narrow-band LED and no dichroic filters. This filter-free approach is applicable to any object with a low level of back-scattering, potentially including trypanosomal parasites, which are close relatives to *Leishmania*; stronger back scattering objects can be studied with a single emission filter (no dichroic mirror is required). The brightness of narrow-band LEDs used in this study was low compared to standard laser or mercury lamp sources, but still allowed registration of the fluorescent signal with cheap cameras, while the more advanced camera did not depend on this limitation. Ongoing progress in LED technologies is expected to bring cheap brighter devices to the market.

Our device can potentially be converted into a TIRF fluorescent illuminator by moving the mirror closer to the periphery of the objective to meet TIRF illumination conditions, and adding an emission filter or a second mirror to remove the reflected illuminating beam. This will open new opportunities for cost-efficient TIRF applications. A similar approach has been demonstrated on a custom-modified microscope with two small mirrors^30^ mounted on a holder in a DIC-slot slot to provide collimated TIRF illumination. BSDF and EF techniques can both be used on the same sample. An example would include combined labeling of the sample by fluorophore- and gold nanoparticles-labeled probes. Such a sample could be studied with the same light source and a single emission filter.

Our device can also convert a transmission light microscope with regular transmission objectives into a surface reflection microscope; such an option is otherwise impossible for microscopes of this class. This finding converts transmission objectives into universal objectives with very broad applicability.

## Methods

### Design of the device

Our device was designed to fit a Nikon Eclipse TE2000U microscope DIC objective slot, 16 mm (width) by 50 mm (length) by 3.6 mm (height). The microscope was equipped with Nikon CFI Plan Fluor series objectives of 5-, 40- and 100 × magnification. Schematic of the device was generated with Google SketchUp.

### Materials

Rod mirrors were from Edmund optics. Emission filters, a single mode and multi-mode optical fibres (25, 50 μm fibre core), fibre-coupled warm white light and narrow-band (365, 470 nm) LEDs were from Thorlabs. 632 nm laser was from Melles Griot (model # 05-LHP-991). Green fluorescent polystyrene beads and Quantum dots QD_655_ were from Life Technologies. 100 nm gold nanospheres were from Nanopartz. Smaller gold nanoparticles were from BBI International. Polystyrene nanospheres were from Polysciences.

### Cameras

USB3 EM-CCD iXon Ultra 888 camera (Andor Technologies, Belfast, UK), USB2 5 Mpixel CMOS DK5.1P camera (Aptina, San Jose, USA), and colour 0.3 Mpixel analogue WAT-221S 1/2″ camera (Watec, Yamagata-Ken, Japan) connected to a PC via a USB frame grabber were used in this study to collect images.

### Image collection and processing

Gold and polystyrene nanoparticles were non-specifically bound to Piranha etch (1:3 (volume/volume) mixture of 30%hydrogen peroxide in water with sulfuric acid) cleaned coverslips in tunnel slides, where coverslips were separated from slides with 100 μm double-sided sticky tape spacers. Images for Fig. 1C,E, inset, 2, 3, 4A-E, and 4 F-G, 7 C, D-H were collected in series of 20–40 frames with Andor and Watec cameras respectively, averaged, and background corrected with ImageJ. Images for Figs 5,6 and 8 were collected with Aptina camera as single frames, background-corrected and median-filtered with ImageJ. The rest of the images were collected with conventional digital cameras. Theoretical point spread function of the microscope was generated with ImageJ plugin Mosaic PSF Tool 2D. The modulus transform functions (MTFs) were calculated with a custom written MATLAB program, where 44 × 44 pixel ROIs were background subtracted, normalized, and the modulus of the Fourier transform of the ROI was then plotted parallel and perpendicular to the axis of the rod mirror of the inserted device. Theoretical MTFs were generated using NA 1.33 (water) and 655 nm wavelength for EF mode, NA 1.3 and 480 nm for BSDF and NA 0.5 and 600 nm for FSDF.

### *Malaria* cells preparation

Merozoites of *Malaria falciparum* were cultured in human type O erythrocytes as described previously^31^, air-dried and methanol-fixed to slides. Prior to use dried blood smears were mounted in isotonic solution.

### *Leishmania* cells preparation

Promastigotes of *Leishmania mexicana* (WHO strain MNYC/BZ/62/M379) were grown, prepared and DAPI stained as published^32^.

**Unicellular green alga** was collected from a natural water sample.

**DHB technique.** The bilayers were formed as described^25^.

### Photolithography of *Silicon* wafers

Following a standard photolithography protocol^33^, silicon wafers were spin coated with SU-8 2 photoresist, soft baked, exposed to near UV light through a photomask, post exposure baked to cross link the exposed SU-8, then developed with SU-8 developer.

## Additional Information

**How to cite this article**: Ishmukhametov, R. R. *et al.* A simple low-cost device enables four epi-illumination techniques on standard light microscopes. *Sci. Rep.* **6**, 20729; doi: 10.1038/srep20729 (2016).
